# Metastatic epithelioid trophoblastic tumor of the lung: A case report

**DOI:** 10.1097/MD.0000000000010306

**Published:** 2018-04-20

**Authors:** Wangjun Lei, Fangbiao Zhang, Chunhui Zheng, Chun Zhao, Shaosong Tu, Yingwei Bao

**Affiliations:** a Department of Radiology, Suichang People's Hospital, Suichang; b Department of Cardiothoracic Surgery, Zhejiang University, Lishui Central Hospital, Lishui, Zhejiang, P.R. China.

**Keywords:** epithelioid trophoblastic tumor, lung, metastatic

## Abstract

**Rationale:**

Epithelioid trophoblastic tumor (ETT) is a very rare form of gestational trophoblastic disease (GTD) which arises from neoplastic proliferation of intermediate trophoblasts. Metastatic ETT of the lung is extremely rare in postmenopausal women.

**Patient concerns:**

Here we describe a 50-year-old woman with a metastatic ETT of the lung showing increasing tracer uptake at PET/CT.

**Diagnosis:**

Hematoxylin–eosin staining showed a tumor composed of nests of epithelioid cells with necrotic debris and peritumoral hyaline-like material. Immunohistochemical staining of the tumor cells was positive for human chorionic gonadotropin (HCG) and cytokeratin 18.

**Interventions:**

The patient underwent thoracoscopic lower left lobectomy combined with mediastinal lymphadenectomy. At surgery, a solid mass (size 3.0 × 3.0 cm) was found in the left lower lung.

**Outcomes:**

The patient was discharged on the tenth day postsurgery, following an uneventful recovery. Three months postsurgery, the patient was asymptomatic and is currently being managed with close follow-up.

**Lessons:**

Metastatic ETT of lung is a very rare disease. Complete surgical resection and chemotherapy may be the critical therapeutic option.

## 1 Introduction

Epithelioid trophoblastic tumor (ETT) is a rare variety of gestational trophoblastic disease (GTD) that is composed of intermediate trophoblast cells, which was initially described by Shih and Kurman in 1998.^[1]^ Site of the lesion in 50% cases is in the lower uterine segment and cervix, 30% in the uterine corpus, and rarely in lungs, brain, spine.^[2]^ It usually occurs in reproductive-age women between 1 and 18 years following a previous gestation.^[3]^ According to the reports, approximately 100 cases have been reported till now.^[2]^ Metastases are reported in 25% of cases.^[4]^ Because of the rarity, the biologic behavior, imaging characteristics, and therapeutic schedule are not fully established. Herein, we report a rare case of a metastatic ETT of the lung in a 49-year-old postmenopausal woman. The clinical, radiological, immunohistochemical analyses, and a review of previous cases are also presented.

## 2 Case presentation

**Figure 1:**
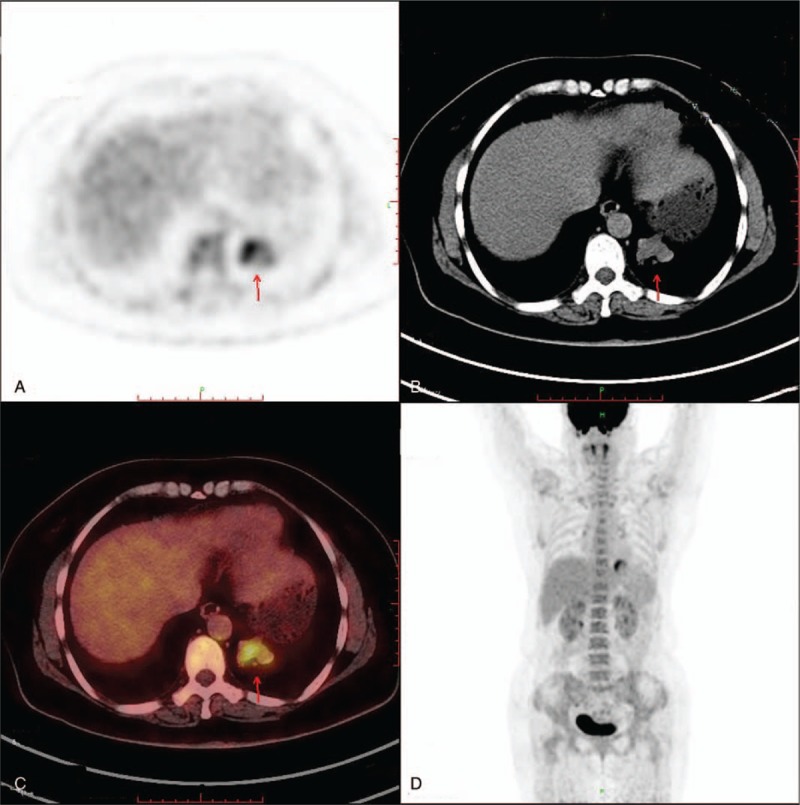
A computed tomography (CT) scan of the chest revealed a 3.0 × 3.0 cm left lung mass. Maximum intensity projection PET (D) and axial (B) CT, corresponding PET (A), and fused (C) images showed FDG uptake of the lesion (arrow) with SUVmax of 6.5.

**Figure 2:**
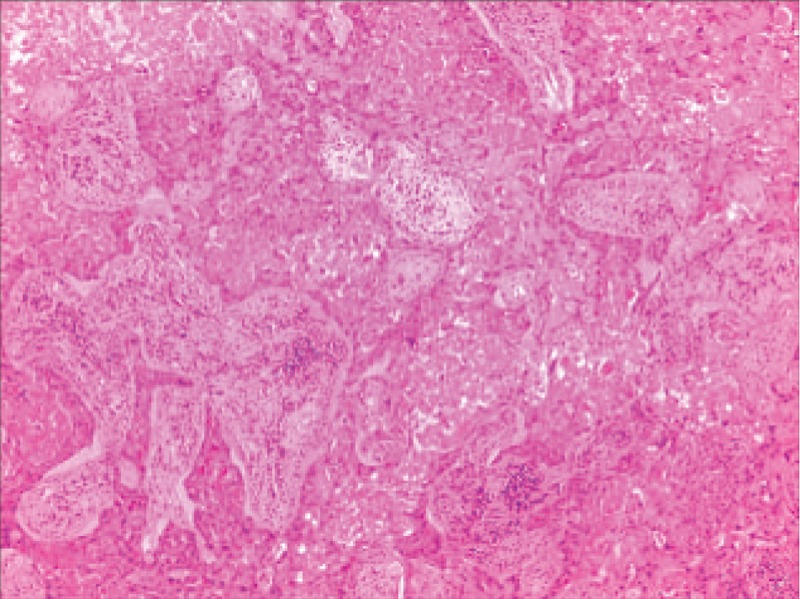
Low-power photomicrograph (A, hematoxylin–eosin, original magnification ×40) revealed the tumor cells in nests and the area of geographic hyalinizing necrosis.

**Figure 3:**
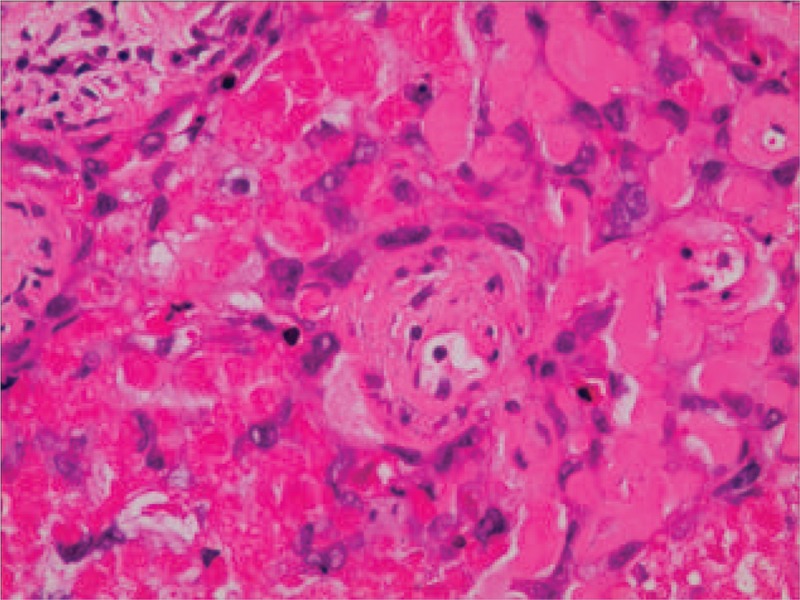
High-power photomicrograph (B, hematoxylin–eosin, original magnification ×200) showed epithelioid cells with necrotic debris and peritumoral hyaline-like material.

**Figure 4:**
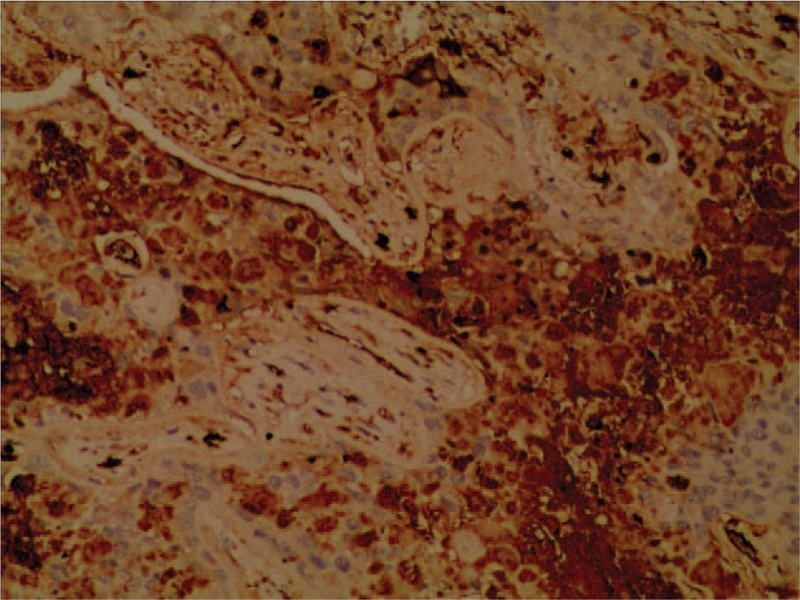
Immunohistochemical staining of the tumor cells was strongly positive for human chorionic gonadotropin (HCG) (C, original magnification ×100).

A 49-year-old asymptomatic female (gravida 1, para 1, and 7 years postmenopausal) presented to the Lishui Hospital, Zhejiang University (Lishui, China) for a physical examination. Informed consent was obtained from the patient for the publication of this case. The patient had a history of hydatidiform mole and no history of diabetes mellitus, hypertensive disease, cigarette smoking, or coronary disease. The serum concentrations of Na^+^, K^+^, Ca^2+^, Mg^2+^, Cl^−^, and glucose were all within the normal limits. A computed tomography (CT) scan of the chest revealed a 3.0 × 3.0 cm left lower lung mass. Other examinations, including blood routine examination, coagulation function, electrocardiogram, lung functional examination, and transesophageal echocardiogram were normal. Due to the possibility that the mass was malignant, the positron emission tomography/computed tomography (PET/CT) was performed. A PET/CT scan of the chest revealed a 3.0 × 3.0 cm left lower lung mass. Maximum intensity projection PET (D) and axial (B) CT, corresponding PET (A), and fused (C) images showed fluorodeoxyglucose (FDG) uptake of the lesion (arrow) with standard uptake value (SUV) max of 6.5 (Fig. 1). Distant metastasis was not found during PET/CT. A video-assisted thoracoscopic surgery (VATS) was performed under general anesthesia on August 17, 2017. At surgery, a solid mass (size 3.0 × 3.0 mm) was found in the left lower lung. Low-power photomicrograph (original magnification ×40) revealed the tumor cells in nests and the area of geographic hyalinizing necrosis (Fig. 2). High-power photomicrograph (original magnification ×200) showed epithelioid cells with necrotic debris and peritumoral hyaline-like material (Fig. 3). Immunohistochemical staining of the tumor cells was positive for human chorionic gonadotropin (HCG) (original magnification ×100) (Fig. 4). The patient was discharged on the tenth day postsurgery, following an uneventful recovery. Three months postsurgery, the patient was asymptomatic and is currently being managed with close follow-up.

## 3 Discussion

ETT is a rare form of GTD arising from neoplastic proliferation of intermediate trophoblasts distinct from choriocarcinoma and placental site trophoblastic tumor (PSTT), which was recognized as a distinct entity in the late 1990s.^[1]^ The intermediate trophoblast is thought to have a phenotype that is between that of the primitive cytotrophoblast and the terminally differentiated syncytiotrophoblast.^[5]^ Common sites of ETT are lower uterine segment and cervix (50%) and uterine corpus (30%), and rarely in lungs, spine, vagina, fallopian tube, broad ligament, gallbladder, and small bowel.^[2]^

Patients with uterine ETT present with different symptoms that are dependent on tumor location, including vaginal bleeding, amenorrhea, abdominal pain, and abdominal bloating. Patients with metastatic pulmonary ETT have dyspnea or hemoptysis as the primary symptoms.^[6]^ In our case, patient did not complain of symptom. Due to nonspecific clinical features, the diagnosis of metastatic pulmonary ETT is easily delayed. Imaging examinations, including CT or MRI, are beneficial for assessing ETT.^[7]^ The PET/CT may helpful to differentiate ETT from other pulmonary tumors. A slightly increased SUVmax via FDG-avidity helps in differentiating ETT from other tumors.^[3]^ In our cases, the PET/CT was performed, and revealed a 3.0 × 3.0 cm left lower lung mass and showed FDG uptake of the lesion with SUVmax of 6.5.

The differential diagnosis of ETT includes other gestational trophoblastic neoplasias, such as choriocarcinoma and PSTT. β-hCG may be play a critical role to distinguish it from other tumors. In contrast to choriocarcinoma, serum β-hCG is usually elevated in the lower range (<2500 mIU/mL).^[4]^ Immunohistochemical staining is essential to make an accurate diagnosis. Both immunohistochemical and morphologic studies showed that the ETT is mainly composed of chorionic-type intermediate trophoblastic cells.^[1]^ These cells are arranged in nests, cords, sheets, and trabeculae in a background of prominent hyaline material and geographic necrosis.^[6]^ Immunohistochemical staining of the tumor cells showed patchy reactivity for cytokeratin 18, pancytokeratin, epithelial membrane antigen, human placental lactogen (hPL), β-hCG, and p63.^[8]^

To date, because of the rare occurrence of this neoplasm, there is no established standard treatment for metastatic pulmonary ETT. However, according to the report, complete surgical resection still is the critical therapeutic option for extrauterine ETT.^[6]^ According to the Kim et al,^[6]^ chemotherapy is one of the treatment options for patients who had incomplete surgical resection, recurrence, metastasis of tumor. The regimens of chemotherapy such as MAC (methotrexate, actinomycin, and chlorambucil) or EMACO (etoposide, methotrexate, actinomycin D, cyclophosphamide, and vincristine) were commonly used.^[9–12]^ But curative effect of chemotherapy is unknown. So a prospective randomized control study between surgery and chemotherapy should by performed by collaborative multicenter or national data collection to provide powerful and crucial evidence for deciding the optimal treatment.

Postoperative surveillance to detect metastasis and recurrence of ETT remains a huge challenge. However, Long-term follow-up with monthly serum β-hCG and hPL tests was recommended due to its clinical features and laboratory findings. In our cases, she received complete surgical resection. Chemotherapy was not performed because of low level of β-hCG (1.6 mIU/mL, range 0–5 mIU/mL). Three months postsurgery, the patient was disease-free and is currently being managed with close clinical and radiologic follow-up.

## 4 Conclusion

It is significant to correctly identify this neoplasm. Our purpose of the case report is to heighten the awareness for clinicians that ETT may arise within unusual locations. As more cases get reported, there will be more clarity regarding the diagnostic strategies, treatment schedules, and prognostic factors of metastasis in ETT.

## Author contributions

Drafted the manuscript: Wangjun Lei.

Performed the surgery: Shaosong Tu, Fangbiao Zhang, Chunhui Zheng, Chun Zhao.

Helped collect clinical data and made critical revisions for important intellectual content: Yingwei Bao.

All authors read and approved the final manuscript.

**Data curation:** Chunhui Zheng, Shaosong Tu.

**Methodology:** Chun Zhao.

**Resources:** Fangbiao Zhang.

**Writing – original draft:** Wangjun Lei.

**Writing – review and editing:** Yingwei Bao.
